# Dendritic spine head diameter is reduced in the prefrontal cortex of progranulin haploinsufficient mice

**DOI:** 10.1186/s13041-024-01095-5

**Published:** 2024-06-05

**Authors:** Anna K. Cook, Kelsey M. Greathouse, Phaedra N. Manuel, Noelle H. Cooper, Juliana M. Eberhardt, Cameron D. Freeman, Audrey J. Weber, Jeremy H. Herskowitz, Andrew E. Arrant

**Affiliations:** Center for Neurodegeneration and Experimental Therapeutics, Alzheimer’s Disease Center, Evelyn F. McKnight Brain Institute, Department of Neurology, University of Alabama at Birmingham, Birmingham, AL USA

**Keywords:** Progranulin, Frontotemporal Dementia, Dendritic spines, Social dominance, Prefrontal cortex

## Abstract

Loss-of-function mutations in the progranulin (*GRN*) gene are an autosomal dominant cause of Frontotemporal Dementia (FTD). These mutations typically result in haploinsufficiency of the progranulin protein. *Grn*^*+/–*^ mice provide a model for progranulin haploinsufficiency and develop FTD-like behavioral abnormalities by 9–10 months of age. In previous work, we demonstrated that *Grn*^*+/–*^ mice develop a low dominance phenotype in the tube test that is associated with reduced dendritic arborization of layer II/III pyramidal neurons in the prelimbic region of the medial prefrontal cortex (mPFC), a region key for social dominance behavior in the tube test assay. In this study, we investigated whether progranulin haploinsufficiency induced changes in dendritic spine density and morphology. Individual layer II/III pyramidal neurons in the prelimbic mPFC of 9–10 month old wild-type or *Grn*^*+/–*^ mice were targeted for iontophoretic microinjection of fluorescent dye, followed by high-resolution confocal microscopy and 3D reconstruction for morphometry analysis. Dendritic spine density in *Grn*^*+/–*^ mice was comparable to wild-type littermates, but the apical dendrites in *Grn*^*+/–*^ mice had a shift in the proportion of spine types, with fewer stubby spines and more thin spines. Additionally, apical dendrites of *Grn*^*+/–*^ mice had longer spines and smaller thin spine head diameter in comparison to wild-type littermates. These changes in spine morphology may contribute to altered circuit-level activity and social dominance deficits in *Grn*^*+/–*^ mice.

**Supplementary Information:**

The online version contains supplementary material available at 10.1186/s13041-024-01095-5.

Heterozygous loss-of-function mutations in progranulin (*GRN*) are a major autosomal dominant cause of Frontotemporal Dementia (FTD), a disease characterized by language or behavior impairments [1, 2]. Patients with FTD due to *GRN* mutations (FTD-*GRN*) develop frontotemporal lobar degeneration with TAR DNA-binding protein 43 (TDP-43) pathology type A, which is characterized by TDP-43 aggregation and neuronal loss, particularly in layer II/III of the cortex [3]. Mutations in *GRN* typically result in haploinsufficiency of progranulin [1, 2], a secreted pro-protein that localizes to lysosomes and has important roles in regulating inflammation, promoting neuronal outgrowth, and maintaining lysosomal function [4, 5]. Haploinsufficiency of progranulin protein, and the resulting loss of its protective effects, are thought to drive FTD-*GRN* pathogenesis. People with loss-of-function mutations on both *GRN* alleles, resulting in complete progranulin deficiency, develop the lysosomal storage disorder Neuronal Ceroid Lipofuscinosis (NCL) [6, 7].

*Grn*^*+/–*^ mice model progranulin haploinsufficiency and are a genetic model of FTD-*GRN. Grn*^*+/–*^ mice develop age-dependent behavioral abnormalities in the 3-chamber sociability, conditioned fear, open field, and marble burying tests [8]. *Grn*^*+/–*^ mice also develop a low social dominance phenotype in the tube test at 9 months of age [9], which can be reversed by restoring progranulin to the mPFC [10]. *Grn*^*–/–*^ mice, which are a genetic model of NCL due to *GRN* mutations, develop most of the same behavioral deficits as *Grn*^*+/–*^ mice, but also develop inflammation and lysosomal abnormalities that model changes in patients with NCL and FTD due to *GRN* mutations [8, 11]. Interestingly, *Grn*^*–/–*^ mice do not develop social dominance deficits in the tube test [9].

Social dominance behavior in the tube test is dependent on a circuit involving the mediodorsal thalamus (MD) and medial prefrontal cortex (mPFC) [12]. In this MD-mPFC circuit, excitatory neurons project from the MD to mPFC layer II/III pyramidal neurons, primarily targeting the apical dendrites [13]. *Grn*^*+/–*^ mice have impaired conductivity along MD to mPFC projections [14], and we have reported that *Grn*^*+/–*^ mice have decreased dendritic arborization of layer II/III pyramidal neurons [9]. To further characterize changes to mPFC neuronal morphology that might impact thalamocortical circuit activity in *Grn*^*+/–*^ mice, we investigated dendritic spine density and morphology on mPFC layer II/III pyramidal neurons.

Dendritic spines are the postsynaptic site for the majority of excitatory synapses, and are classically organized into thin, mushroom, or stubby spines, and filopodia [15]. Dendritic spine morphology is closely tied to function [15, 16], so examining how progranulin haploinsufficiency influences spine morphology may help to elucidate the mechanisms underlying low social dominance in *Grn*^*+/–*^ mice. To test how progranulin haploinsufficiency affects dendritic spine morphology of layer II/III pyramidal neurons in the mPFC, we performed high-resolution imaging and morphometry analysis of individual dye-filled layer II/III pyramidal neurons in the prelimbic cortex (Fig. 1B).

**Fig. 1:**
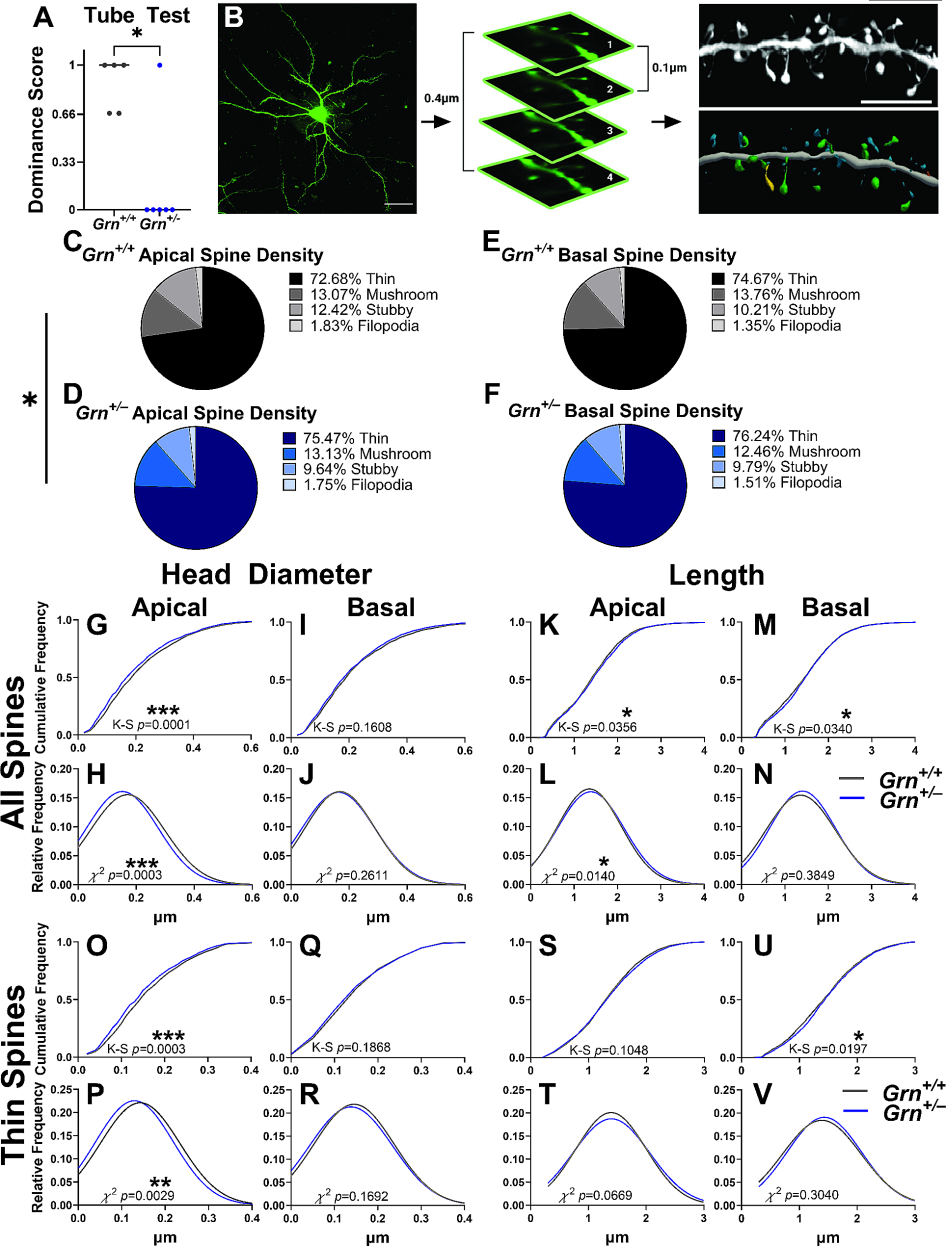
Dendritic spine morphology changes in *Grn*^*+/–*^ mice. **A**) *Grn*^*+/–*^ mice showed a low-dominance phenotype in the tube test at 10 months of age (*n* = 5–6 mice, 2–3 mice of each sex per group, two-tailed Mann-Whitney U test, *p* = 0.0368). **B)** Representative 20X image of a Lucifer yellow injected neuron (scale bar represents 100 μm) and imaging parameters. Representative 60X dendritic segment with its corresponding reconstruction (scale bar represents 5 μm). Thin spines are shown in blue, mushroom spines in green, stubby spines in orange, filopodia in yellow. **C–F)** The distribution of spine types on apical dendrites of *Grn*^*+/–*^ mice was shifted relative to *Grn*^*+/+*^ mice (**C, D**, chi-square, *p* = 0.0186) but there was no difference in the distribution of spine types on basal dendrites between genotypes (**E, F**, chi-square, *p* = 0.3208). **G–J**) *Grn*^*+/–*^ mice had decreased dendritic spine head diameter on apical dendrites (**G**, K-S test, *p* = 0.0001, **H**, chi-square, *p* = 0.0003), but not on basal dendrites (**I**, K-S test, *p* = 0.1608, **J**, chi-square, *p* = 0.2611). **K–N**) *Grn*^*+/–*^ mice showed increased spine length on apical dendrites (**K**, K-S test, *p* = 0.0356, **L**, chi-square, *p* = 0.014). On basal dendrites, analysis of cumulative frequency distribution (**M**, K-S test, *p* = 0.034), though not relative frequency distribution (**N**, chi-square, *p* = 0.3849) indicated an increase in spine length. **O-R**) *Grn*^*+/–*^ mice had reduced thin spine head diameter on apical dendrites (**O**, K-S test, *p* = 0.0003, **P**, chi-square, *p* = 0.0029), but not basal dendrites (**Q**, K-S test, *p* = 0.1868, **R**, chi-square, *p* = 0.1692). **S-V**) *Grn*^*+/–*^ mice did not exhibit a significant increase in thin spine length on the apical dendrites (**S**, K-S test, *p* = 0.1048, **T**, chi-square, *p* = 0.0669). On basal dendrites, analysis of cumulative frequency distribution (**U**, K-S test, *p* = 0.0197), but not relative frequency distribution (**V**, chi-square, *p* = 0.3040) revealed an increase in thin spine length. *n* = 2175–3325 thin spines, 336–370 mushroom spines, 280–541 stubby spines, 40–68 filopodia per apical or basal dendrite for each genotype, from 5–6 mice per genotype. Relative frequency distributions are shown as Gaussian curve fits. * = *p* < 0.05, ** = *p* < 0.01, *** = *p* < 0.0001

First, we performed the tube test assay to confirm the low social dominance phenotype in the 10-month-old *Grn*^*+/–*^ mice and *Grn*^*+/+*^ littermates used for this study (Fig. 1A). Next, we performed iontophoretic microinjections of Lucifer yellow dye into individual layer II/III neurons of the mPFC (Fig. 1B, Table S1). Then, we used confocal microscopy to capture 60x images of the apical and basal dendrites (Table S1). Raw confocal images underwent deconvolution and were imported into Neurolucida 360 for morphometric analysis (Fig. 1B) [17].

Dendritic spine density among apical or basal dendrites was comparable in *Grn*^*+/+*^ and *Grn*^*+/–*^ mice (Fig. S1A, B). However, there was a shift in the distribution of individual spine types on apical dendrites of *Grn*^*+/–*^ mice, with a shift toward fewer stubby and more thin spines (Fig. 1C, D). Spine type distribution on basal dendrites was comparable between the two genotypes (Fig. 1E, F).

We next analyzed the morphology of spines on apical and basal dendrites. *Grn*^*+/–*^ mice had longer dendritic spines on both apical and basal dendrites (Fig. 1K-N). Analysis of spine types revealed that thin spines from *Grn*^*+/–*^ mice were longer on the basal dendrites, but not apical dendrites (Fig. 1S-V). Mushroom and stubby spine length were comparable in *Grn*^*+/–*^ mice and littermate controls on both apical and basal dendrites (Fig. S1E, F, I, J).

*Grn*^*+/–*^ mice exhibited an overall reduction of spine head diameter on apical (Fig. 1G, H), but not basal (Fig. 1I, J) dendrites. Analysis of spine types revealed a decrease in apical thin spine head diameter (Fig. 1O, P), but no significant changes to apical mushroom or stubby spine head diameter (Fig. S1C, G). There was no difference in head diameter of thin (Fig. 1Q, R), mushroom (Fig. S1D), or stubby (Fig. S1H) spines on basal dendrites of *Grn*^*+/–*^ mice versus wild-type littermates. For most measures, progranulin genotype had a similar effect across sex (Fig. S2A-E) except for basal spine length (Fig. S2F-J), which was driven by changes in female mice (Fig. S2G, I, J).

In this study, we focused on *Grn*^*+/–*^ mice due to the lack of a social dominance phenotype in *Grn*^*–/–*^ mice [10, 11]. *Grn*^*–/–*^ mice also do not develop the impaired thalamocortical conductance observed in *Grn*^*+/–*^ mice, though both *Grn*^*–/–*^ and *Grn*^*+/–*^ mice exhibit impaired excitability of mediodorsal thalamic neurons and signs of impaired cortical circuitry [14]. It is therefore not clear if the gene-dose effect observed for phenotypes such as lysosomal dysfunction and inflammation in *Grn*^*+/–*^ and *Grn*^*–/–*^ mice would be observed for changes to mPFC dendritic spines. Reduced spine density has been reported in CA1 of *Grn*^*–/–*^ mice [18, 19], though this effect may vary by background strain and has not been consistently observed in C57Bl/6 mice [11, 19], the strain of mice used in this study.

These data show subtle changes in dendritic spine morphology in *Grn*^*+/–*^ mice that are consistent with reports of impaired dendritic arborization [9] and impaired thalamocortical conductance [14]. Together, these data provide structural and physiological data indicating impaired MD-mPFC circuit function. Activity in the mPFC and synaptic strength of the MD-mPFC circuit drive social dominance behavior in the tube test [12]. Thus, impaired MD-mPFC circuit activity could be a mechanism driving low social dominance in *Grn*^*+/–*^ mice [9, 12]. Patients with FTD-*GRN* also exhibit abnormal thalamocortical connectivity, with preclinical hyperconnectivity followed by loss of connectivity as disease progresses [20]. The altered length and head diameter of apical spines in *Grn*^*+/–*^ mice could contribute to impaired mPFC activity, as spine morphology is closely associated with function. Spine neck length and head shape influence diffusion of signaling molecules, including calcium [15]. Thus, spine morphology can impact synaptic strength by regulating calcium flux, amplitude, and duration as well as availability of receptors at the post-synaptic density (PSD) [15]. In humans, decreased thin spine head diameter in the dorsolateral PFC is correlated with impaired cognition [21]. There might be a similar effect in *Grn*^*+/–*^ mice, where reduced thin spine head diameter contributes to impaired mPFC activity in *Grn*^*+/–*^ mice, which could contribute to impaired social dominance behavior.

Several mechanisms could explain the altered dendritic spine morphology we observed in *Grn*^*+/–*^ mice. The impaired thalamocortical conductance of *Grn*^*+/–*^ mice [14] could reduce the excitation/inhibition ratio of layer II/III neurons and result in smaller spine head diameter by dampening synaptic calcium flux [16]. Progranulin haploinsufficiency might also directly impact spine morphology through loss of progranulin’s neurotrophic effects [4]. Alternatively, loss of progranulin’s functions in lysosomes may impact membrane dynamics, receptor localization at the PSD, and vesicle recycling, all of which depend on lipid regulation and the endolysosomal pathway [22].

In summary, this report provides further evidence of abnormal neuronal morphology in layer II/III mPFC neurons of progranulin haploinsufficient mice. Together with other studies [9, 14], these data are consistent with impaired thalamocortical input to the mPFC as a potential mechanism of impaired social dominance in *Grn*^*+/–*^ mice.

### Electronic supplementary material

Below is the link to the electronic supplementary material.

Supplementary Material 1

## Data Availability

All data generated and analyzed in this study is presented in the main text or additional information files. Source data is available from the corresponding author on request.

## Abbreviations

*GRN*: Progranulin
FTD: Frontotemporal Dementia
NCL: Neuronal Ceroid Lipofuscinosis
mPFC: medial prefrontal cortex
MD: mediodorsal thalamus
TDP-43: TAR DNA-binding protein 43
PSD: postsynaptic density
