# Supplementary material for: Dendritic spine head diameter is reduced in the prefrontal cortex of progranulin haploinsufficient mice
Source: Mol Brain. 2024 Jun 5;17:33. doi: 10.1186/s13041-024-01095-5 (PMC11155153; doi:10.1186/s13041-024-01095-5)
Supplement: Supplementary file 1 — Supplementary Material 1 [file 13041_2024_1095_MOESM1_ESM.docx]

**Supplementary Material for:**

**Dendritic spine head diameter is reduced in the prefrontal cortex of progranulin haploinsufficient mice**

Anna K Cook, Kelsey M Greathouse, Phaedra N Manuel, Noelle H Cooper, Juliana M Eberhardt, Cameron D Freeman, Audrey J Weber, Jeremy H Herskowitz, Andrew E Arrant

Center for Neurodegeneration and Experimental Therapeutics, Alzheimer’s Disease Center, Evelyn F. McKnight Brain Institute, Department of Neurology, University of Alabama at Birmingham, Birmingham, AL, USA

**Materials and Methods**

**Figure S1**

**Figure S2**

**Table S1**

**Materials and Methods**

**Animals**

Male and female *Grn^+/­–^* mice (1) (Jackson Laboratory #036771) on a congenic C57Bl6/J background were compared to wild type littermates in the tube test and dendritic spine analysis (n = 2-3 mice of each sex per genotype). Mice were bred and housed in a facility accredited by the Association for Assessment and Accreditation of Laboratory Animal Care, with a 12 hour light/dark cycle. Mice had free access to food (Envigo #7917) and water. All protocols were approved by University of Alabama at Birmingham’s Institutional Animal Care and Use Committee.

**Tube Test**

As previously described (2), Mice were paired against non-cage mate, same sex mice of the opposite genotype (*Grn^+/­+^* vs *Grn^+/­–^).* Testing was carried out in a soundproof, negative pressure room designed for behavioral assays. The mice were habituated to the room for 1 hour before testing, but received no habituation to the tube prior to their first match. A clear plastic tube (30.3 cm long, 3.2 cm diameter) was placed on a bench and the cages containing the desired mice were moved near the tube before each match. The mice for each pairing were identified and moved to either end of the tube at the same time. The mice were gently encouraged to enter the tube, releasing their tails once all four paws entered the tube. The mice were allowed to freely interact in the tube, and the first mouse to place two paws outside of the tube was considered the “loser” of the match. Tests lasting longer than 2 minutes were stopped and re-run again at the end of the session. Testing occurred over 3 rounds, with each mouse facing a different mouse of the opposite genotype in each round. Dominance score was calculated by dividing the number of wins by the number of matches for each mouse.

**Perfusions and Tissue Processing**

Mice were processed for iontophoretic dye injection as previously described (3). Mice were anesthetized with Euthasol (MWI Animal Health, Catalog #O11344), then transcardially perfused using a peristaltic pump (Cole Parmer) with cold 1% paraformaldehyde (PFA, Sigma Aldrich, Catalog #P6148) for 1 minute, then with cold 4% PFA with 0.125% glutaraldehyde (Fisher Scientific, Catalog #BP2547) for 10 minutes. Immediately after perfusion, the brain was extracted and drop fixed in 4% PFA with 0.125% glutaraldehyde at 4 °C for 8–12 hours. Once fixed, brains were coronally sliced into 250 μm sections on a Leica vibratome (VT1000S, speed 70, frequency 7) in cold 0.1 M phosphate buffer (PB). Each section was stored at 4 °C in an individual well of a 48 well plate in 0.1 M PB with 0.1% sodium azide (Fisher, Catalog #BP922I).

**Iontophoretic Microinjections**

Iontophoretic microinjections were performed as previously described (3). Injections were visualized using a Nikon Eclipse FN1 upright microscope on an air table. Microinjections were performed with micropipettes (A-M Systems, Catalog #603500) pulled at time of use and filled with 2 µL of 8% Lucifer yellow dye (ThermoFisher, Catalog #L453). Sliced brains were incubated in 1x PBS and Hoechst 33347 (Life Technologies, Catalog #H1399) for 5 minutes at room temperature prior to injection. Microinjections were then performed on layer II/III neurons using 2 nA of negative current to fill the neuron with Lucifer yellow over 5 minutes. Neuron selection was blind, and if the entire neuron did not fill with dye the neuron was not used in the experiment. Multiple neurons per hemisphere were injected for each brain. After injection, the tissue was carefully placed on a glass slide with two 125 µm spacers (Electron Microscopy Sciences, Catalog #70327-20S) and coverslipped using Vectashield (Vector Labs, Catalog #H1000) and No. 1.5 coverslips (Warner, Catalog #64-0716). Injected tissue was stored in the dark at 4 °C.

**Confocal Microscopy**

Dendrite imaging was performed as previously described (3). A blinded experimenter imaged injected neurons on a Nikon Ti2 C2 confocal microscope using a Plan Apo 60x/1.40 NA oil-immersion objective. Z-stacks were obtained from secondary dendrites from dye-filled neurons that met the following criteria: 1) within 80 µm working distance of microscope, 2) relatively parallel with the surface of the section, 3) located between 40 and 120 µm from the soma, and 4) no overlap with other branches. Nikon Elements 4.20.02 image capture software was used to collect z-stacks with a step size of 0.1 µm, image size of 1024 × 512 pixels, zoom of 4.8x, line averaging of 4x, and acquisition rate of 1 frame/second.

**Dendritic Spine Analysis**

Dendritic spine analysis was performed as previously described (3). Z-stacks of dendrites acquired on the confocal were deconvolved using Huygens Deconvolution System (16.05, Scientific Volume Imaging) with the following settings: deconvolution algorithm: GMLE; maximum iterations: 10; signal to noise ratio: 15; quality: 0.003. Deconvolved images were saved in .tif format. Image stacks were then imported into Neurolucida 360 (2.70.1, MBF Biosciences, Williston, Vermont). Dendrites were traced using the semi-automatic directional kernel algorithm, excluding the outer 5 µm of each dendrite. Assigned points were verified to ensure they matched the dendrite in X, Y, and Z planes, and adjusted if necessary. A voxel-clustering algorithm was used to reconstruct the dendritic spines and used the following parameters: outer range = 5 µm, minimum height = 0.3 µm, detector sensitivity = 80%, minimum count = 8 voxels. The spines produced from the semi-automatic reconstruction were examined to verify that all identified spines were real and no spines were excluded. If necessary, the detector sensitivity was increased to include any missing spines and merge and slice tools were used to correct errors in the morphology of each spine. Using constant parameters, each spine was automatically classified as a thin spine, mushroom spine, stubby spine, or filopodium. Dendrite reconstruction images were exported to Neurolucida Explorer (2.70.1, MBF Biosciences, Williston, Vermont), in which they underwent branched structure analysis. Spine density was calculated as the number of spines per 10 µm of dendrite per apical or basal dendritic arbor and averaged by mouse.

**Statistical Analysis**

Data were graphed and statistically analyzed using GaphPad Prism 10.0.2 (GraphPad Software, La Jolla, CA). Tube test dominance score was analyzed by a two-tailed Mann-Whitney U test. Apical and basal dendritic spine density was calculated as an average value for each mouse and graphed as mean ± SEM. Spine density was first tested for normality using the Anderson-Darling, D’Agostino-Pearson omnibus, and Shapiro-Wilk tests. As all spine density measurements passed the normality tests, the data were analyzed using an unpaired, two-tailed *t*-test. Spine type distributions were for each genotype were graphed as the percentage of all spines classified as either thin, mushroom, stubby, or filopodia. Spine type distributions were analyzed using a Chi-square test on the actual numbers of spines for each genotype. Measures of spine length and head diameter were graphed as a cumulative and relative frequency distributions of individual spines from each genotype. Cumulative frequency distributions were analyzed by Kolmogorov-Smirnov test, and relative frequency distributions were analyzed by chi-square test. For relative frequency distributions, spines were binned into 0.3 µm increments for length or 0.05 µm increments for head diameter, and chi-square analysis was performed on the total numbers of dendrites in each bin. For all statistical tests, apical and basal arbors were analyzed separately, and significance was defined as *p* < 0.05.

**References**

1. Martens LH, Zhang J, Barmada SJ, Zhou P, Kamiya S, Sun B, et al. Progranulin deficiency promotes neuroinflammation and neuron loss following toxin-induced injury. J Clin Invest; 2012. p. 3955-9.

2. Arrant AE, Filiano AJ, Warmus BA, Hall AM, Roberson ED. Progranulin haploinsufficiency causes biphasic social dominance abnormalities in the tube test. Genes, Brain, and Behavior 2016;15:588-603.

3. Weber AJ, Adamson AB, Greathouse KM, Andrade JP, Freeman CD, Seo JV, et al. Conditional deletion of ROCK2 induces anxiety-like behaviors and alters dendritic spine density and morphology on CA1 pyramidal neurons. Mol Brain. 2021;14(1):169.

**
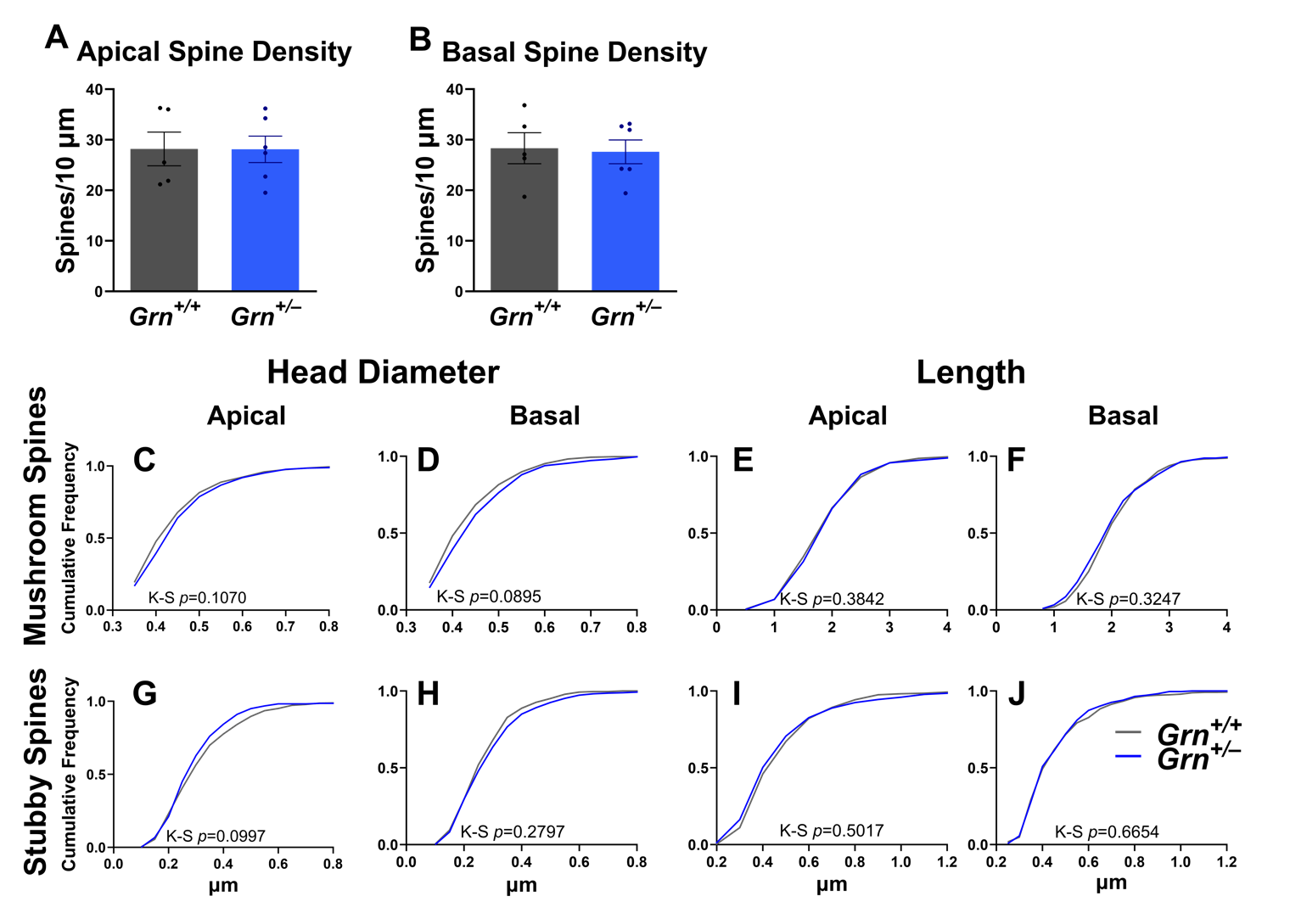
Supplementary Figure 1. No differences in dendritic spine density or in head diameter and length of other spine types. A, B)** We observed no difference in spine density on apical dendrites (**A**, unpaired *t*-test, *p* = 0.9865) or basal dendrites (**B**, unpaired *t*-test, *p* = 0.8531) between wild-type and *Grn^+/–^* mice. **C, D)** There were no significant differences in mushroom spine head diameter on apical dendrites (**C**, K-S, *p* = 0.101) or basal dendrites (**D**, K-S, *p* = 0.0895) between wild type and *Grn^+/–^* mice. **E, F)** *Grn^+/–^* mice did not have a difference in mushroom spine length on apical dendrites (**E**, K-S, *p* = 0.3842) or basal dendrites (**F**, K-S, *p* = 0.3247). **G, H)** *Grn^+/–^* mice did not have a significant difference in stubby spine head diameter on apical dendrites (**G**, K-S, *p* = 0.0997) or basal dendrites (**H**, K-S, *p* = 0.2797). **I, J)** We found no differences in stubby spine length on apical dendrites (**I**, K-S, *p* = 0.5017) or basal dendrites (**J**, K-S, *p* = 0.6654) between wild type and *Grn^+/–^* mice. n = 280–541 stubby spines, n = 336–570 mushroom per dendritic arbor for each genotype, from 5–6 mice per genotype.

**
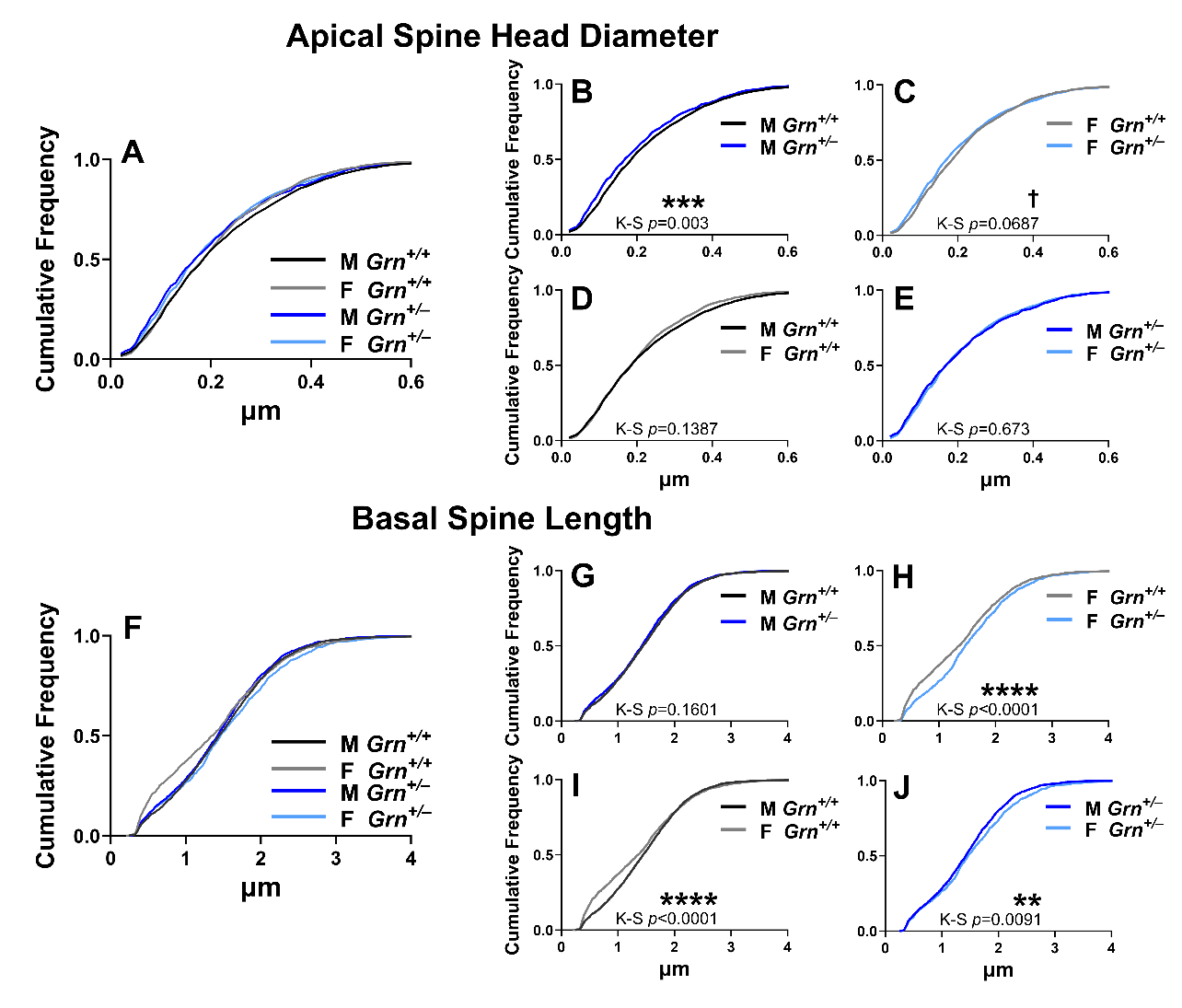
Supplementary Figure 2. Absence and presence of sex differences in representative morphometric parameters. A)** Absence of sex differences in apical spine head diameter (n = 2–3 female mice per genotype, n = 3 male mice per genotype). **B, C)** Reduction in apical spine head diameter is maintained in *Grn^+/–^* male mice (**B**, K-S, *p*=0.003) and trending in female *Grn^+/–^* mice (**C**, K-S, *p*=0.0687). **D, E)** There was no difference in apical spine head diameter between male and female *Grn^+/+^* mice (**D**, K-S, *p*=0.1387) or between male and female *Grn^+/–^* mice (**E**, K-S, *p*=0.673). **F)** Sex differences in basal spine length (n = 2–3 female mice per genotype, n = 3 male mice per genotype). **G, H)** Male *Grn^+/–^* mice did not have longer basal spines than male *Grn^+/+^*  mice (**G,** K-S, *p*=0.1601) but female *Grn^+/–^*  mice had longer basal spines than female *Grn^+/+^*  mice (**H**, K-S, *p*<0.0001) **I)** Male and female *Grn^+/+^*  mice had significantly different basal spine length (**I**, K-S, *p*<0.0001), **J)** as did male and female *Grn^+/–^* mice (**J**, K-S, *p*=0.0091). n = 2175–3325 thin spines, 336–370 mushroom spines, 280–541 stubby spines, 40–68 filopodia per apical or basal dendric arbor, for each genotype, from 5–6 mice per genotype. Relative frequency distributions are shown as Gaussian curve fits. † = *p*<0.1, * = *p*<0.05, ** = *p*<0.01, *** = *p*<0.0001.

| **Mouse** | **Sex** | **Neurons Injected** | **Apical Dendrites** | **Basal Dendrites** |
| --- | --- | --- | --- | --- |
| *Grn^+/+^* | M | 9 | 9 | 9 |
|  | F | 5 | 5 | 2 |
|  | F | 6 | 6 | 6 |
|  | M | 10 | 10 | 10 |
|  | M | 6 | 6 | 6 |
| *Grn^+/–^* | F | 4 | 4 | 3 |
|  | M | 5 | 5 | 4 |
|  | F | 4 | 4 | 2 |
|  | M | 5 | 4 | 5 |
|  | M | 5 | 1 | 5 |
|  | F | 5 | 5 | 3 |

**Supplementary Table 1. Description of the mice used in this study and the number of neurons imaged per mouse.** 4–10 neurons were injected per mouse and at least 1 apical and 1 basal dendrite of each neuron were imaged.
